# Agentic artificial intelligence in inflammatory bowel disease: toward autonomous and adaptive care

**DOI:** 10.1093/crocol/otag066

**Published:** 2026-06-27

**Authors:** Animesh Acharjee, Daniela Santos

**Affiliations:** Cancer and Genomic Sciences, School of Medical Sciences, College of Medicine and Health, University of Birmingham Dubai, Dubai, 341799, UAE; Cancer and Genomic Sciences, School of Medical Sciences, College of Medicine and Health, University of Birmingham, Edgbaston, B15 2TT, United Kingdom; Centre for Health Data Research, University of Birmingham, Birmingham, B15 2TT, United Kingdom; Institute of Translational Medicine, University Hospitals Birmingham NHS, Foundation Trust, United Kingdom; Cancer and Genomic Sciences, School of Medical Sciences, College of Medicine and Health, University of Birmingham Dubai, Dubai, 341799, UAE

**Keywords:** IBD, Agentic AI, Machine Learning

## Abstract

Inflammatory bowel disease (IBD) is a chronic, heterogeneous condition requiring ongoing monitoring and iterative therapeutic adjustment. Current management relies on multiple clinical, biochemical, imaging, and molecular data sources that are collected intermittently and rarely integrated into a unified representation of disease activity, leading to reactive clinical decision-making. Although artificial intelligence (AI) has demonstrated promise in IBD, most applications remain task-specific and operate on data collected at isolated time points, failing to capture the longitudinal nature of disease. Agentic AI offers a shift toward continuous and adaptive care by integrating diverse data streams into an evolving representation of disease state. This enables early detection of change and supports proactive intervention through a closed-loop system linking monitoring, interpretation, and action. Despite its potential, challenges related to data quality, interpretability, and clinical integration must be addressed for implementation.

## From reactive to longitudinal IBD care

Inflammatory bowel disease, comprising Crohn’s disease and ulcerative colitis, is driven by complex interactions between immune dysregulation, environmental factors, host–microbiome dynamics, and treatment effects. Disease activity evolves over time, contributing to substantial heterogeneity in clinical behaviour and therapeutic response. Despite advances in biologic therapies, this variability continues to limit effective management.

In clinical practice, disease assessment remains fragmented. Decisions rely on inflammatory biomarkers (example: C-reactive protein, fecal calprotectin), imaging, endoscopy, and patient-reported outcomes (PROs), each capturing only a partial and time-specific view of disease activity. As a result, management is largely reactive, with treatment adjustments occurring after clinical deterioration rather than in anticipation of disease progression.^1^

AI has been increasingly applied in IBD to support specific tasks such as endoscopic scoring and prediction of treatment response.^2^ While these approaches demonstrate strong performance, they remain confined to individual clinical tasks and are not embedded within longitudinal care. Predictions are generated at isolated time points without accounting for disease evolution or cumulative treatment effects. The limitation of current AI in IBD is not accuracy, but its inability to operate within a continuous decision-making framework.

## Agentic AI and continuous disease representation

Agentic AI provides a framework to address this limitation by enabling continuous integration of data, updating representations of disease state, and aligning decisions with clinical objectives. Rather than functioning as standalone tools, these systems link monitoring, interpretation, and intervention within an ongoing process.

A key requirement for adaptive care is the ability to maintain an evolving representation of patient status. In IBD, this is challenging due to heterogeneity and the fragmentation of clinical, molecular, and patient-generated data. Agentic systems address this by integrating these inputs into a continuously updated model, often conceptualized as a digital twin.^3^^,^^4^ This patient-specific representation is iteratively refined as new data become available, allowing disease trajectories to be tracked rather than inferred from isolated measurements.

This enables anticipation of disease evolution. For example, variability in biologic pharmacokinetics may lead to gradual loss of response despite stable dosing. Integrating drug levels, anti-drug antibodies, and inflammatory markers longitudinally could allow earlier detection of these shifts and support proactive adjustment before clinical deterioration. In this way, disease assessment becomes dynamic rather than static.

## Closed-loop care and longitudinal decision-making

Building on this continuous representation, agentic AI enables a shift toward longitudinal, closed-loop care. In current practice, decisions are made at discrete time points, often triggered by symptoms or delayed biomarker changes. Even when predictive tools exist, they are typically used to support individual clinical decisions rather than continuous longitudinal disease management.

Closed-loop systems link monitoring, interpretation, and intervention into a unified process.^5^ As new data are incorporated, disease activity is reassessed, treatment response reevaluated, and recommendations updated. This allows early signals, such as subclinical inflammation or gradual loss of response, to be identified and acted upon before overt relapse.

This enables more dynamic management. Longitudinal patterns in biomarkers and patient-reported outcomes can inform earlier intervention, while sustained remission may support treatment de-escalation. Decisions are therefore based on trajectories rather than single measurements.

Rather than relying solely on predefined thresholds, these systems refine decision strategies based on accumulated patient data and prior treatment outcomes. This introduces an adaptive component in which management evolves alongside the disease course.

## Agentic AI implementation: learning from another domain

A proof-of-concept for this approach exists outside IBD in the management of sepsis. The AI Clinician model used reinforcement learning to learn dynamic treatment strategies for intravenous fluids and vasopressors in critically ill patients, updating recommendations according to evolving patient states and observed outcomes.^5^ Although this system was not designed for autonomous deployment, it demonstrates the feasibility of modeling clinical care as a sequential decision-making problem in which patient state, therapeutic action and outcome are linked over time.^6^ The principles underlying this framework are directly applicable to IBD, where management similarly depends on repeated reassessment of disease activity, treatment exposure, and therapeutic response. In IBD, an analogous agentic system could integrate symptoms, biomarkers, drug levels, imaging, endoscopy and molecular data to recommend monitoring intensity, therapy optimization, escalation, de-escalation or referral within a clinician-supervised loop.

In practice, such systems would likely rely on layered data acquisition strategies depending on resource availability and disease severity. A pragmatic longitudinal dataset could include symptoms, treatment exposure, C-reactive protein and fecal calprotectin collected at baseline and repeated at defined intervals after treatment initiation or optimization (for example 8-12 weeks and subsequently every 3-6 months during maintenance).^1^ More advanced implementations could incorporate therapeutic drug monitoring, intestinal ultrasound, endoscopy, histology and emerging molecular profiling when available.^7^ Rather than requiring complete multimodal data at every time point, agentic systems could dynamically adapt to missing or lower-resolution inputs, enabling deployment across settings with variable access to specialist investigations.

Such frameworks may be particularly valuable in regions with limited access to IBD specialists, therapeutic drug monitoring, advanced imaging or repeated endoscopy. As the global burden of IBD continues to rise, many healthcare systems face increasing demand alongside substantial disparities in specialist availability and longitudinal disease monitoring.^8^ In these settings, agentic AI systems could support standardized treat-to-target strategies through integration of noninvasive and remotely acquired data, including symptoms, patient-reported outcomes, C-reactive protein, fecal calprotectin and treatment exposure history. A layered monitoring strategy could enable lower-cost, high-frequency surveillance using accessible biomarkers, while reserving resource-intensive investigations such as intestinal ultrasound, endoscopy, histology or molecular profiling for patients identified as high risk or demonstrating probable loss of response. Rather than replacing specialist care, these systems could function as clinician-supervised decision-support frameworks that prioritize patients requiring escalation, support earlier intervention, and reduce delays in therapeutic optimization.

## Longitudinal biomarker integration in agentic IBD care

Agentic AI systems in IBD would rely on continuous integration of longitudinal clinical and biological data to maintain an updated representation of disease activity and therapeutic response. Rather than depending on isolated measurements, these frameworks could incorporate sequential data collected before treatment initiation, after therapy introduction or optimization, during maintenance, and at suspected disease flare or loss of response.

At baseline, the system could integrate disease phenotype, treatment exposure, patient-reported outcomes, inflammatory biomarkers such as C-reactive protein (CRP) and fecal calprotectin, and standard laboratory markers. When available, higher-resolution modalities including intestinal ultrasound (IUS), endoscopy, histology, therapeutic drug monitoring and emerging molecular profiling could further refine risk stratification and prediction of therapeutic response.^7,^^9^

Following treatment initiation or adjustment, reassessment of symptoms, CRP and fecal calprotectin at approximately 8-12 weeks, followed by regular monitoring during maintenance, could support early identification of non-response or evolving loss of response. Additional modalities such as drug levels, IUS or endoscopy could be incorporated selectively depending on disease severity, discordant findings or uncertainty in disease-state estimation.

**Figure 1:**
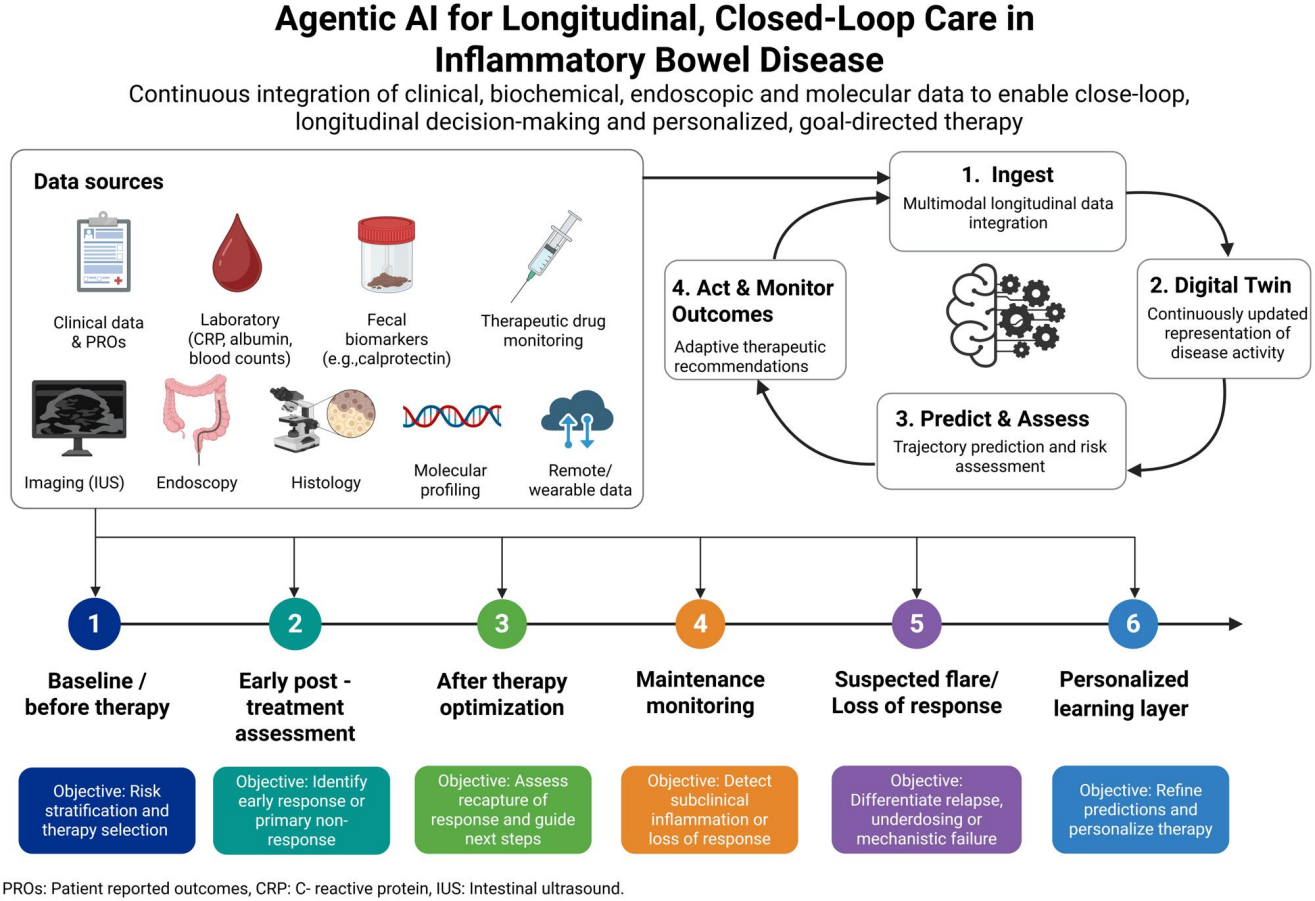
Conceptual framework of agentic AI–enabled longitudinal care in inflammatory bowel disease. Multimodal clinical, biochemical, imaging, endoscopic, histological, molecular and patient-generated data are continuously integrated to create a patient-specific digital twin. This evolving disease representation supports trajectory prediction, risk assessment and adaptive therapeutic recommendations within a closed-loop system. Clinical outcomes and newly acquired data continuously refine future predictions and therapeutic decisions across the patient journey.

Importantly, clinically meaningful decision support may not require complete multimodal data at every time point. A layered approach using accessible biomarkers and remotely acquired patient data could allow deployment across healthcare systems with variable access to IBD specialists, advanced imaging or repeated endoscopy, while still supporting earlier intervention and more standardized treat-to-target care.^10^ A potential framework for longitudinal biomarker integration and adaptive monitoring in agentic IBD care is outlined in Table 1 and Figure 1.

**Table 1:** Proposed longitudinal data inputs for an agentic AI framework in IBD care.

Care phase	Core data inputs	Optional/advanced inputs	Suggested timing	Decision supported
**Baseline/before therapy**	Diagnosis, disease phenotype, extent, severity, prior therapy, symptoms, PROs, CRP, fecal calprotectin, albumin, hemoglobin	Endoscopy, histology, IUS, molecular data, microbiome, transcriptomics	Before treatment initiation	Risk stratification; therapy selection; monitoring intensity
**Early post-treatment assessment**	Symptoms, adherence, adverse events, CRP, fecal calprotectin	Drug level and anti-drug antibodies if biologic therapy; IUS if available	8-14 weeks after starting therapy	Identify primary non-response or early response
**After therapy adjustment**	Symptoms, adherence, CRP, fecal calprotectin	Drug level/antibodies, IUS, endoscopy if discordant or severe	8-12 weeks after dose escalation, switch, or optimization	Assess recapture of response; decide continue vs switch
**Maintenance remission**	Symptoms/PROs, treatment exposure, CRP, fecal calprotectin	IUS; endoscopy/histology at longer intervals or if biomarkers worsen	Symptoms every 1-3 months; biomarkers every 3-6 months	Detect subclinical relapse; support de-escalation or continuation
**Suspected flare/loss of response**	Symptoms, CRP, fecal calprotectin, stool infection testing, medication adherence	Drug level/antibodies, IUS, endoscopy, histology	At flare or biomarker rise	Distinguish inflammation, infection, IBS-like symptoms, underdosing, mechanistic failure
**Long-term/emerging precision layer**	Longitudinal clinical outcomes, treatment history, biomarkers	Genomics, transcriptomics, proteomics, metabolomics, microbiome	Baseline and selected reassessment points; not necessarily every visit	Refine disease trajectory prediction and personalized treatment selection

Abbreviations: PROs, patient-reported outcomes; CRP, C-reactive protein; IUS, intestinal ultrasound.

## Goal-directed therapy and clinical impact

Agentic AI extends the treat-to-target paradigm by embedding therapeutic goals within a continuous decision process.^1^ While current strategies prioritize endpoints such as mucosal healing and sustained remission, they are implemented through intermittent assessments and relatively fixed treatment pathways.

In contrast, agentic systems enable continuous optimization. Treatment can be adjusted in real time to maintain or achieve targets, informed by evolving disease activity and response patterns. This includes early identification of non-response, detection of emerging loss of response, and balancing efficacy with treatment-related risks.

Decision-making is thus guided not only by current disease state but also by response trajectories and the likelihood of achieving long-term outcomes.^10^ Management becomes dynamically tailored rather than protocol driven.

## Challenges and considerations

Despite its potential, implementation of agentic AI in IBD faces several challenges. Data quality, data collection and variability complicate the development of reliable longitudinal models. Many AI systems also rely on associative patterns without capturing underlying biological mechanisms, limiting interpretability.

The adaptive nature of these systems raises additional concerns regarding reproducibility, accountability, and oversight. Ensuring safe translation into clinical practice will require robust validation frameworks and clear data governance structures.^11^

## Conclusion

Current IBD management remains limited by episodic assessment and static prediction, which fail to capture the evolving nature of disease. The key limitation is the inability to engage with disease as a dynamic process over time.

Agentic AI offers a framework for continuous, adaptive care by integrating disease representation, longitudinal decision-making, and goal-directed optimization. This shifts management from reactive intervention toward proactive disease control, aligning treatment with evolving patient needs.

## Data Availability

No new data was generated or analyzed in support of this review article.
